# TNF-α/Stearate Induced H3K9/18 Histone Acetylation Amplifies IL-6 Expression in 3T3-L1 Mouse Adipocytes

**DOI:** 10.3390/ijms25126776

**Published:** 2024-06-20

**Authors:** Fatemah Bahman, Areej Al-Roub, Nadeem Akhter, Ashraf Al Madhoun, Ajit Wilson, Nourah Almansour, Fatema Al-Rashed, Sardar Sindhu, Fahd Al-Mulla, Rasheed Ahmad

**Affiliations:** 1 Immunology & Microbiology Department, Dasman Diabetes Institute, Dasman 15462, Kuwait; fatemah.bahman@dasmaninstitute.org (F.B.); areej.abualroub@dasmaninstitute.org (A.A.-R.); nadeem.akhter@dasmaninstitute.org (N.A.); ajit.wilson@dasmaninstitute.org (A.W.); nourah.almansour@dasmaninstitute.org (N.A.); fatema.alrashed@dasmaninstitute.org (F.A.-R.); sardar.sindhu@dasmaninstitute.org (S.S.); 2 Animal and Imaging Core Facilities, Dasman Diabetes Institute, Dasman 15462, Kuwait; ashraf.madhoun@dasmaninstitute.org; 3 Translational Research Department, Dasman Diabetes Institute, Dasman 15462, Kuwait; fahd.almulla@dasmaninstitute.org

**Keywords:** IL-6, TNF-α/stearate, adipocytes, H3K9/18

## Abstract

Extensive evidence supports the connection between obesity-induced inflammation and the heightened expression of IL-6 adipose tissues. However, the mechanism underlying the IL-6 exacerbation in the adipose tissue remains unclear. There is general agreement that TNF-α and stearate concentrations are mildly elevated in adipose tissue in the state of obesity. We hypothesize that TNF-α and stearate co-treatment induce the increased expression of IL-6 in mouse adipocytes. We therefore aimed to determine IL-6 gene expression and protein production by TNF-α/stearate treated adipocytes and investigated the mechanism involved. To test our hypothesis, 3T3-L1 mouse preadipocytes were treated with TNF-α, stearate, or TNF-α/stearate. IL-6 gene expression was assessed by quantitative real-time qPCR. IL-6 protein production secreted in the cell culture media was determined by ELISA. Acetylation of histone was analyzed by Western blotting. Il6 region-associated histone H3 lysine 9/18 acetylation (H3K9/18Ac) was determined by ChIP-qPCR. 3T3-L1 mouse preadipocytes were co-challenged with TNF-α and stearate for 24 h, which led to significantly increased IL-6 gene expression (81 ± 2.1 Fold) compared to controls stimulated with either TNF-α (38 ± 0.5 Fold; *p* = 0.002) or stearate (56 ± 2.0 Fold; *p* = 0.013). As expected, co-treatment of adipocytes with TNF-α and stearate significantly increased protein production (338 ± 11 pg/mL) compared to controls stimulated with either TNF-α (28 ± 0.60 pg/mL; *p* = 0.001) or stearate (53 ± 0.20 pg/mL, *p* = 0.0015). Inhibition of histone acetyltransferases (HATs) with anacardic acid or curcumin significantly reduced the IL-6 gene expression and protein production by adipocytes. Conversely, TSA-induced acetylation substituted the stimulatory effect of TNF-α or stearate in their synergistic interaction for driving IL-6 gene expression and protein production. Mechanistically, TNF-α/stearate co-stimulation increased the promoter-associated histone H3 lysine 9/18 acetylation (H3K9/18Ac), rendering a transcriptionally permissive state that favored IL-6 expression at the transcriptional and translational levels. Our data represent a TNF-α/stearate cooperativity model driving IL-6 expression in 3T3-L1 cells via the H3K9/18Ac-dependent mechanism, with implications for adipose IL-6 exacerbations in obesity.

## 1. Introduction

Obesity in humans is increasing worldwide by alarming proportions and is characterized by chronic low-grade inflammation or metabolic inflammation, further leading to a multitude of metabolic disorders including hypertension, atherosclerosis, type 2 diabetes (T2D), cardiovascular diseases, liver diseases, and certain types of cancer [1]. The elevated infiltration of activated immune effector cells in the expanding adipose tissue underlies the hallmark signature of metabolic inflammation [2]. The adipose tissue in obesity is marked by elevated expression of pro-inflammatory cytokines, including IL-6, IL-1β, and TNF-α, which leads to impairment in insulin signaling and loss of glycemic control [3]. Notably, adipose IL-6 is attracting attention as an important immune modulator and a key contributor to metabolic inflammation [4]. Increased IL-6 levels have been associated with glycogen inhibition, activation of glycogen phosphorylase, increased lipolysis, and increased triglyceride production, resulting in the development of insulin resistance [5,6]. Individuals with obesity were found to have higher mRNA levels of inflammatory cytokines in their circulating monocytes, compared to lean individuals [7], and the induced hyperlipidemia in mice was associated with expansion in proinflammatory monocyte subpopulations. Lipid-laden monocytes and macrophages show activated phenotypes and the enhanced expression of proinflammatory transcription factors such as NF-kβ, which contributes to the synthesis of reactive oxygen species (ROS) and macrophage migration inhibitory factors, both factors thus playing key roles in inflammation [8].

It is also noteworthy that adipocyte-driven metabolites such as saturated free fatty acids (SFFAs) act as potent mediators of metabolic inflammation and insulin resistance in obesity [9]. Indeed, an independent association was found between increased circulatory FFA levels and the risk for cardiovascular disease or T2D [10,11,12]. Among the SFAs linked to the pathogenesis of obesity/T2D, palmitic acid is well-recognized as a key player in metabolic inflammation [13,14,15], but the role of stearic acid as an inflammatory trigger is more complex, given its ambivalent effects in metabolic conditions. While stearic acid may have some protective effects [16,17], other studies show that it promotes inflammation by enhancing the expression of TNF-α and IL-6 in different cell types [18,19]. Macrophage extravasation into the white adipose tissue in obesity and M1 polarization leads to the expression of proinflammatory cytokines and the development of systemic inflammation in both humans and mice [20]. Among the adipocytokines, both IL-1β and TNF-α have been associated with triggering inflammatory responses and insulin resistance in obesity [21].

Epigenetic modifications are heritable changes in gene expression without involving DNA sequence alterations and refer to the methylation of DNA cytosine residues within cytosine-guanine dinucleotides (CpG islands) and methylating/acetylating histone residues lysine/arginine [22]. The main types of epigenetic modifications include DNA methylation, histone modifications, chromatin remodeling, and ncRNAs. High-fat diets are known to alter gene expression via epigenetic modifications such as DNA methylation and histone post-translational modifications [23]. DNA and histone modifications modify the chromatin structure, thereby regulating the access of transcription factors, while ncRNAs influence gene expression at the RNA level [24]. Experimental and clinical evidence supports the idea that histone modifications play key roles in the development of metabolic disorders [25,26]. Regarding the association between adipose tissue inflammation in obesity and major epigenetic modifications, H3K4/K9m3 and H3K9/K14ac alterations led to increased TNF-α and IL-6 expression and insulin resistance [27,28]. Similarly, another study using both high-fat diet and hyperphagic ob/ob mouse models of obesity, demonstrated that histone H3 lysine 9 and 18 acetylation (H3K9/K18Ac) increased *Tnfa* and *Ccl2* expression in fatty liver at the chromatin level [29]. Notably, H3K4/K9m3 and H3K9/K14ac histone marks were found to be associated with elevated IL-6 expression and induction of metabolic inflammation and insulin resistance [28,30,31]. Further, in regard to IL-6-driving histone modulations, we earlier showed that IL-1β/TNF-α co-stimulations of 3T3-L1 mouse adipocytes and primary human lean adipocytes induced IL-6 expression via the mechanism that involved CREB binding and H3K14 acetylation [32]. We also demonstrated that treating human monocytes with SFA palmitate increased the levels of transcriptionally permissive H3K9/H3K18 acetylation mark at the *Il6* promoter, potentiating the LPS-induced IL-6 production [33]. Given that the role of stearic acid in regulating inflammatory responses in adipocytes remains elusive, we herein tested our hypothesis whether the TNF-α/stearate co-stimulatory challenge upregulated the expression of IL-6 in adipocytes, and our data show that such an immune-metabolic insult promoted the IL-6 production by increasing H3K9/H3K18 acetylation at the *Il6* promoter in 3T3-L1 adipocytes.

## 2. Results

### 2.1. TNF-α and Stearate Act Synergistically to Enhance the IL-6 Gene Expression and Protein Production by Adipocytes

**Figure 1:**
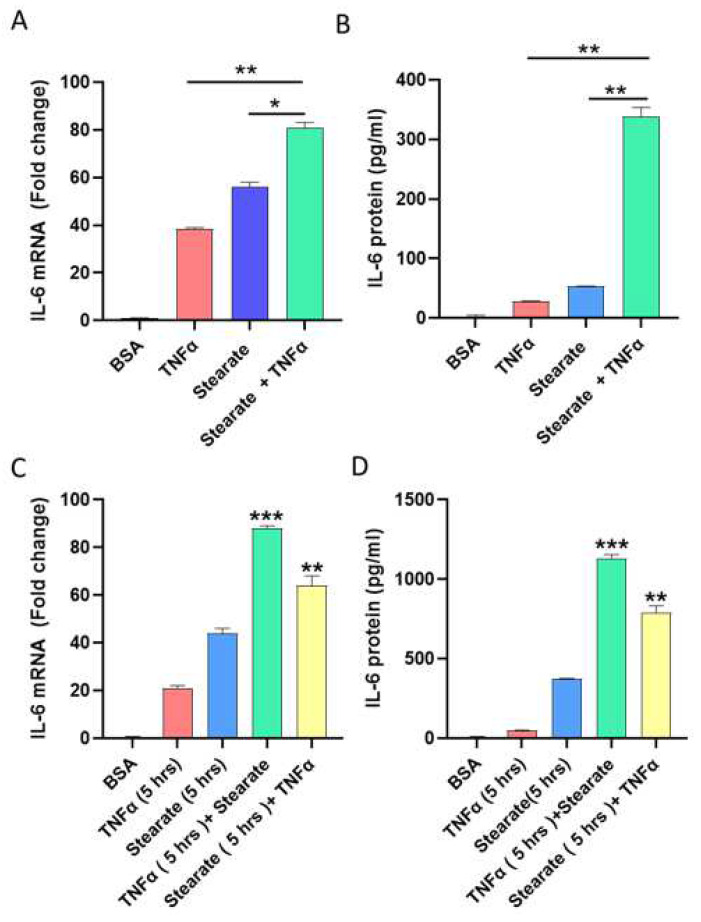
Costimulatory effect of stearate and TNF-α on IL-6 expression in 3T3-L1 mouse adipocytes. 3T3-L1 mouse adipocyte monolayers were stimulated for 24 h with stearate (200 μM) and TNF-α (10 ng/mL), alone or combined. Both cells and culture media were collected. (**A**) Total RNA was extracted from the cells and IL-6 mRNA was quantified by real time RT-PCR. Relative mRNA expression was expressed as a fold change. (**B**) IL-6 protein in culture media was determined by sandwich ELISA. In priming experiments, cells, and supernatants were collected wherein cells were either pre-treated for 5 h with TNF-α and later incubated for 24 h with stearate or pre-treated for 5 h with stearate and later incubated for 24 h with TNF-α. Expression of (**C**) IL-6 mRNA and (**D**) IL-6 protein is shown in 3T3-L1 adipocytes primed with either TNF-α or stearate. Data are expressed as mean ± SEM (n = 3). * *p* < 0.05, ** *p* < 0.01. *** *p* < 0.001.

TNF-α and stearate levels are elevated in obese adipose tissue along with high levels of IL-6 [11,12]. To test whether co-stimulation with stearate and TNF-α could induce and promote IL-6 gene expression and protein secretion by adipocytes, 3T3L-1 adipocytes were challenged with TNF-α and stearate, individually or together. The combined stimulation with TNF-α and stearate resulted in a substantially greater IL-6 production at both mRNA (TNFα: 38 Fold; stearate: 56 Fold; stearate/TNFα: 81 Fold; TNF-α/stearate vs. TNFα: *p* = 0.0025; TNF-α/stearate vs. stearate: *p* = 0.013) and protein production (TNFα: 28.04 pg/mL; stearate: 53.64 pg/mL; stearate/TNFα: 338 pg/mL; TNF-α/stearate vs. TNFα: *p* = 0.001; TNF-α/stearate vs. stearate: *p* = 0.0015) levels (Figure 1A,B). The co-stimulatory effect of TNF-α and stearate on IL-6 gene expression and protein production was greater than the sum of the individual effects of stearate and TNF-α, indicating that a synergistic effect was involved.

To further test whether the stearate had a priming effect, cells were treated with stearate for 5 h before challenging with TNF-α for an additional 24 h. We found that the priming with stearate potentiated TNF-α-induced IL-6 gene expression and protein production. Likewise, the priming with TNF-α for 5 h, followed by a challenge with stearate for 24 h also induced increased IL-6 gene expression and protein production (Figure 1C,D).

### 2.2. Inhibiting Acetylation Reduces the Synergistic Impact of TNF-α/Stearate Co-Stimulation on IL-6 Gene Expression and Protein Production

**Figure 2:**
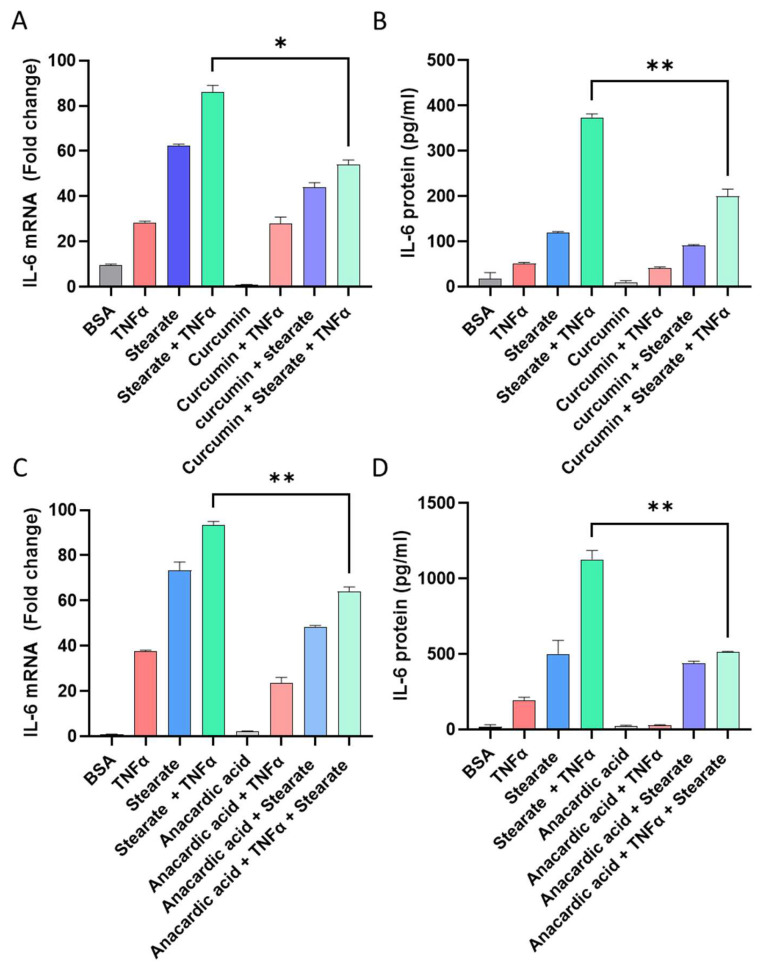
3T3-L1 adipocytes were pre-incubated with curcumin (HATs inhibitor; 20 μM) for 2 h, followed by stimulation with stearate, TNF-α, or TNFα/stearate for 24 h. (**A**) Total RNA was extracted from the cells and IL-6 mRNA was quantified by real-time RT-PCR. (**B**) IL-6 protein was determined by sandwich ELISA. In addition, 3T3-L1 adipocytes were also pre-incubated with anacardic acid (HATs inhibitor; 4 μM) for 1h, followed by stimulation with stearate, TNF-α, or TNFα/stearate for 24 h. (**C**) Total RNA was extracted from the cells and IL-6 mRNA was quantified by real-time RT-PCR. (**D**) IL-6 protein was determined by sandwich ELISA. Data are expressed as mean ± SEM (n = 3). * *p* < 0.05, ** *p* < 0.01.

Regarding the epigenetic mechanism driving amplification of IL-6 in response to co-stimulation by TNF-α and stearate, we tested whether the inhibition of histone acetyltransferases (HATs) impacted the synergistic effect of TNFα/stearate co-stimulation on IL-6 induction. To this end, before treatment with stimulatory agents, adipocytes were pretreated with curcumin, a natural inhibitor, or with anacardic acid, a pharmacological HAT inhibitor that has been shown to inhibit HATs in vitro. [34,35]. Notably, curcumins significantly reduced the IL-6 mRNA expression (*p* = 0.0013) and protein production (*p* = 0.0048) from cells treated with TNFα in combination with stearate (Figure 2A,B). Similarly, anacardic acid significantly reduced the IL-6 gene expression *(p* = 0.0071) and protein production (*p* = 0.0044) from cells treated with TNFα in combination with stearate (Figure 2C,D).

### 2.3. Pharmacological Induced Acetylation Substitutes the Effect of TNF-α or Stearate in the Synergistic Interaction between TNF-α and Stearate for Promoting IL-6 Gene Expression and Protein Production

**Figure 3:**
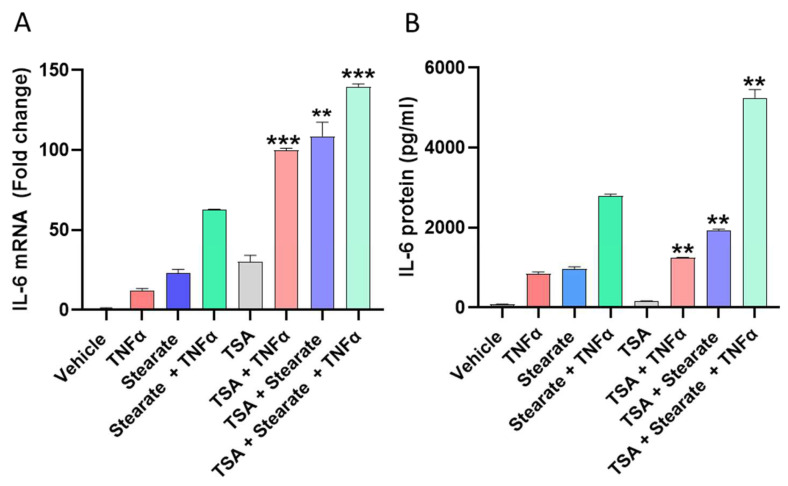
Trichostatin A (TSA) accentuates the synergy between stearate and TNF-α for promoting IL-6 expression. 3T3-L1 adipocytes were treated with TSA (100 nM) for 20 h before stimulation with vehicle, stearate, TNF-α, or TNFα/stearate for 24 h. IL-6 mRNA and protein were determined by qRT-PCR and ELISA, respectively (**A**,**B**). Data were expressed as mean ± SEM. ** *p* < 0.01, *** *p* < 0.001.

To confirm the role of histone acetylation in this synergy, we examined whether the activation of histone acetyltransferases (HATs) modulated the cooperative interaction between TNF-α and stearate for induction of IL-6 gene expression and protein production. Trichostatin A (TSA) is an HDAC inhibitor and plays a significant role in increasing histone acetylation and gene transcription [36]. In order to investigate whether TSA could promote IL-6 gene transcription and protein production, 3T3-L1 adipocytes were treated with TSA prior to the co-treatment with TNFα/stearate. The data show that TSA acted as a substitutive agent for stearate or TNF-α in this synergy, thereby accentuating the cooperativity between stearate and TNF-α for the induction of IL-6 gene expression and protein production (Figure 3A,B).

### 2.4. TNF-α/Stearate Co-Stimulation Increases Il6 Promoter H3K9 and H3K18 Acetylation in 3T3-L1 Adipocytes

**Figure 4:**
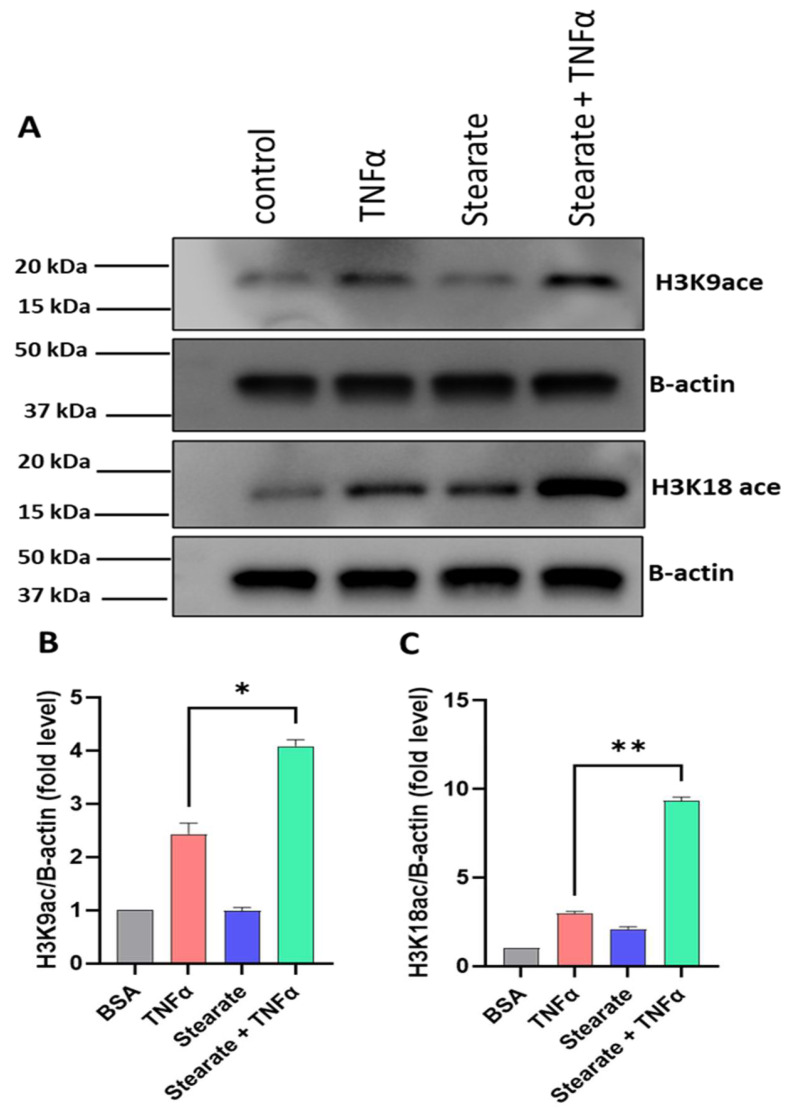
TNF-α and stearate combined treatment enriches histone acetylation. (**A**) 3T3-L1 adipocytes were treated with TNF-α and stearate, alone or in combination. (**A**) Histone acetylation levels were detected by Western blot and compared between treatments, against control. (**B**,**C**) Quantification data of Western blots are shown. Data are presented as mean ± SEM. * *p* < 0.05, ** *p* < 0.01.

Activation of gene expression involves the transcription factors recruiting histone acetyltransferases (HATs) to facilitate acetylation of specific histone residues, ultimately resulting in the opening of chromatin. In contrast, gene expression is suppressed as histones undergo deacetylation by histone deacetylases (HDACs). We next addressed whether TNF-α or stearate affected the acetylation status of histone H3. In this regard, we found that TNF-α/stearate co-treatment caused hyperacetylation only at histone H3K9 and H3K18 residues in 3T3-L1 adipocytes (Figure 4A–C). Since H3K9 hyperacetylation was associated with Histone deacetylase 1 (HDAC1) and HDAC3 depletion [37], we performed experiments and identified that HDAC1 and HDAC3 were reduced in the presence of TNF-α, stearate, or TNF-α/stearate. These results suggest that hyperacetylation induced by TNF/stearate is because of HDAC1 and HDAC3 reduction. TNF-α and stearate work together to regulate acetylation through the depletion of HDAC1 and HDAC3 (Supplementary Figure S1A). Stearate individually shows less increase in H3K9 ace. Since p-JNK increased H3K9 ace [38] and our data show that stearate increased the phosphorylation of JNK, suggesting that stearate may increase H3K9 ace, in part, via p-JNK in the presence of TNF-α (Supplementary Figure S1B).

**Figure 5:**
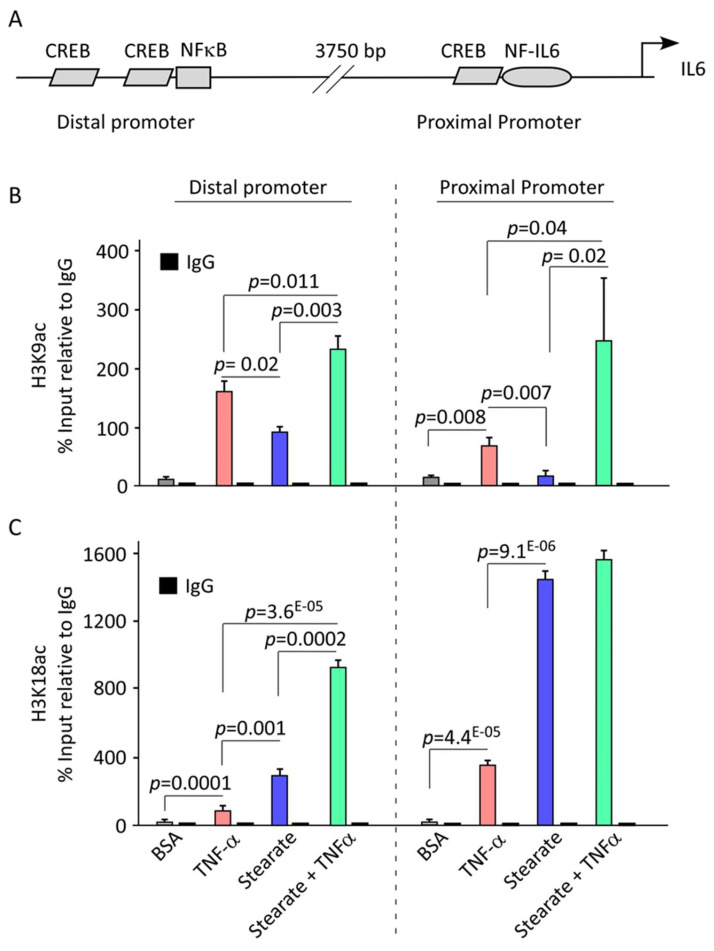
TNF-α and stearate treatments enrich histone acetylation at the IL-6 promoter. 3T3-L1 adipocytes were treated with TNF-α and stearate, alone or in combination. (**A**) Schematic showing the proximal and distal regions of IL-6 promoter for TFs binding sites. The chromatins from adipocytes treated with TNF-α and stearate, alone or in combination, were subjected to ChIP assay using antibodies against (**B**) H3K9 or (**C**) H3K18 followed by qPCR. Data are presented as mean ± SEM (n = 3).

Since TNF-α and stearate, individually and together, increase H3K9 and H3K18 acetylation, we investigated their role in chromatin remodeling of IL-6 promoter in adipocytes, using chromatin immunoprecipitation (ChIP), followed by qPCR. IL-6 regulation involves two regions spanning the first 6 Kb upstream of the transcription start site (Figure 5A). Compared to the control group, TNF-α and stearate treatments resulted in enhanced binding of acetylated H3K9 (Figure 5B) and H3K18 (Figure 5C) to these regulatory regions, indicating active transcription. Notably, TNF-α treatment mediated induced a stronger association of binding of H3K9 antibodies to both proximal and distal IL-6 promoter regions compared to stearate (Figure 5B). Alternatively, stearate exhibited a greater effect on H3K18 antibody recruitment to the IL-6 promoter (Figure 5C). Furthermore, the combined treatment of TNF-α and stearate significantly elevated the binding of the acetylated histones at both regulatory loci, suggesting a synergistic cooperative effect (Figure 5B,C). Taken together, these results indicate that H3K9 and H3K18 acetylation are strongly associated with this cooperativity between TNF-α and stearate for upregulated Il6 gene transcription.

## 3. Discussion

IL-6 emerges as a key player in the pathogenesis of metabolic inflammation in obesity. IL-6 is highly expressed by adipocytes in obese adipose tissue [4], but the underlying mechanism is incompletely understood. In this study, we identified that TNF-α and stearate co-stimulation amplified the IL-6 inflammatory gene transcription and protein expression in adipocytes. It is also notable that stearate, TNF-α, and IL-6 are found elevated in the circulation as well as in adipose tissue in obese states [39,40]. IL-6 is produced by various cells including monocytes, macrophages, and adipocytes under the influence of various stimuli. It has been reported that saturated fatty acid palmitate induces IL-6 in monocytic cells [33]. However, compared with other saturated fatty acids, stearic acid was found to induce the largest amount of IL-6 levels in monocytic cells [18]. Similarly, increasing evidence shows that TNF-α induces IL-6 expression in a broad range of cells, including glioma cells, osteoblasts, and vascular smooth muscle cells, employing different transduction pathways [41,42,43]. Since both TNF-α and stearate are co-elevated in obesity, our findings suggest a new paradigm supporting induction and promotion of metabolic inflammation whereby a cooperative engagement between TNF-α and stearate leads to upregulation of IL-6 expression in adipocytes. This mutually cooperative interaction between the TNF-α and stearate is critical because it has the potential to trigger the expression of inflammatory cytokine IL-6 in adipocytes, implying that targeting each of the inducers in the adipose tissue may have a therapeutic significance in curbing metabolic inflammation and insulin resistance.

Our findings highlighting the TNF-α/stearate cooperativity as a key driver of IL-6 expression are consistent with the following previous reports: (1) the co-existence of two inflammatory agents such as IL-1β and TNF-α in the same milieu of adipose tissue promotes the induction of IL-6 in adipocytes [32]; (2) TNF-α and stearate interaction potentiates the expression of MIP-1α or CCL3 in monocytic cells which has implications for obesity-associated inflammation [12]; and (3) palmitate further promotes the LPS-induced IL-6 expression in monocytic cells [33]. Together, these observations imply that the interactions between inflammatory cytokines and saturated free fatty acids, such as the TNF-α/stearic acid cooperativity per our recent study, may exacerbate adipose inflammation by amplifying IL-6 expression as an underlying mechanism that could feed chronic inflammation in the setting of obesity.

**Figure 6:**
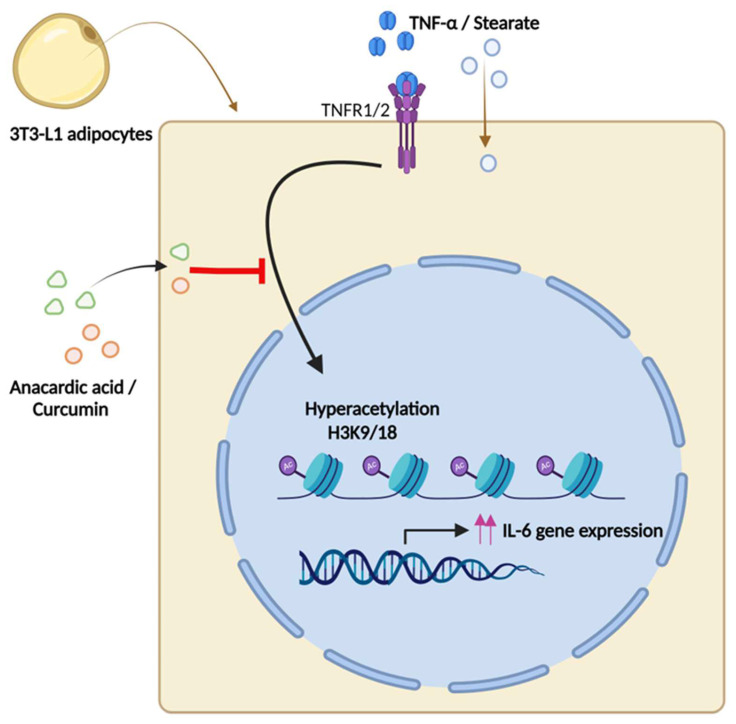
This illustration summarizes our findings. This illustration was created with Biorender.com.

Moreover, our study unveils a regulatory mechanism of epigenetic modulation wherein H3K9 and H3K18 acetylation orchestrates the cooperative interaction between TNF-α and stearate to promote the expression of IL-6 in a mouse adipocyte model, representing a promising therapeutic strategy for targeting metabolic inflammation. Notably, the modification of histone acetylation is crucial for gene expression; hyperacetylation of histone residues enhances gene expression, while deacetylation of these agents represses/silences the transcriptional activity [44,45]. Our investigation revealed that in 3T3L adipocytes, co-stimulation with TNF-α and stearate induced hyperacetylation, specifically at histone H3K9 and H3K18 residues. While IL-6 is primarily regulated by a proximal promoter located within 1.2 KB of the start of transcription, a report by Samuel et al. [46] identified an additional transcriptionally active region located 5Kb upstream. Notably, DNA sequence analysis revealed that both loci accommodate transcription factor consensus sites for transcription factors like CREB, NF-kB, and NF-IL-6. Our study revealed that TNF-α/stearate co-treatment resulted in enhanced histone acetylation at positions K9 and K18 at the two IL-6 regulatory regions, indicating the active transcription. Of note, TNF-α treatment promoted H3K9 remodeling, while Stearate treatment enriched H3K18 at these promoters. As expected, the inhibition of global acetylation reduced the synergistic effect of TNF-α/stearate co-stimulation on IL-6 gene expression (Figure 6).

We previously demonstrated that TNF-α and IL-1β signaling regulated the proximal IL-6 promoter [32], as well as that H3K9 and H3K18 acetylation was crucial for IL-6 gene transcription under the influence of LPS and saturated fatty acid palmitate [33]. While the data obtained using a standard mouse adipocyte model can provide valuable insights into unraveling molecular mechanisms involved, caution will be warranted extrapolating these findings to disease pathophysiology attributes in humans. Therefore, complementary studies using human adipocytes, both from visceral and subcutaneous origins, will next be required to validate and extend our findings.

## 4. Materials and Methods

### 4.1. Cell Culture

3T3-L1 mouse preadipocytes were purchased from the American Type Culture Collection (ATCC) and cultured in Dulbecco’s Modified Eagle’s medium (Gibco, Life Technologies, Grand Island, NY, USA) containing 10% FBS (Gibco, Life Technologies, Grand Island, NY, USA), 2 mM glutamine (Gibco, Invitrogen, Grand Island, NY, USA) and 1% penicillin-streptomycin (Gibco, Life Technologies, Grand Island, NY, USA) in 6-well plates (Costar, Corning Incorporated, Washington, DC, USA) in a humidified incubator at 37 °C under 5% CO_2_ tension. Adipocytes were cultured for 24–36 h until they reached around 70% confluency. At about 70% confluence, cultured adipocytes were stimulated for 24h with stearate (200 μM/mL; Sigma, St. Louis, MO, USA), TNF-α (10 ng/mL; Sigma, St. Louis, MO, USA) or Vehicle (2% BSA). Afterward, cells were harvested and the total protein and RNA were extracted for subsequent analyses.

### 4.2. Real-Time Quantitative RT-PCR (qPCR)

RNeasy mini kit was used for total RNA isolation, following instructions by the manufacturer (Qiagen, Valencia, CA, USA). High-capacity cDNA reverse transcription kit was used to synthesize cDNA from 1 μg of total RNA (Applied Biosystems, Foster City, CA, USA) [47]. TaqMan Gene Expression Assay kits were used to amplify cDNA template for each real-time PCR reaction using two primers (mouse IL-6: Mm00446190_m1; mouse GAPDH:Mm99999915_g1), one TaqMan MGB probe (6-FAM dye-labeled), TaqMan^®^ Gene Expression Master Mix (Applied Biosystems, Foster City, CA, USA), on 7500 Fast Real-Time PCR System (Applied Biosystems, Foster City, CA, USA). GAPDH mRNA expression was used to normalize the target mRNA expression, calculated using the 2^−ΔΔCT^ method. Relative target gene mRNA expression was denoted as fold change relative to the average of control gene expression taken as 1 [48].

### 4.3. ELISA

The levels of IL-6 secretion were measured using ELISA, manufactured by R&D Systems (Minneapolis, MN, USA) following the manufacturer’s instructions. Briefly, 100 uL of diluted IL-6 capture antibody was added to each well of a microplate, and each plate was sealed and incubated overnight at 4 °C. The plates were washed 3 times with wash buffer. Then, 300 µL of reagent diluent was added to each well and the plates were incubated at room temperature for 1 h. After blocking, 100 µL of each sample or standard was added to appropriate wells and the plates were kept at room temperature for 2 h. The plates were washed three times before adding 100 µL of TMB substrate solution to each well, followed by adding 50 µL of stop solution. After washing, 100 µL of IL-6 diluted detection antibody was added to each well and the plates were incubated for 2 h at room temperature. Plates were washed three times before adding 100 µL of streptavidin-HRP solution to each well followed by adding 100 μL of substrate solution to each well. After adding stop solution, absorbance was read at 450 nm with a reference wavelength of 540 nm. The minimum levels of detection were 0.7 pg/mL.

### 4.4. Chromatin Immunoprecipitation (ChIP) Assay

SimpleChIP^®^ Plus Enzymatic Chromatin IP Kit was used to perform ChIP assays, following instructions by the manufacturer (Cell Signaling Technology Inc., Danvers, MA, USA) [49]. Briefly, 3T3-L1 mouse preadipocytes were differentiated into adipocytes and treated with agents as required. The cells were then crosslinked with 4% formaldehyde (Sigma, Taufkirchen, Germany) to yield 200–800 bp chromatin fragments and the digested chromatin fragments were immunoprecipitated using provided primary antibodies specific to the p65 subunit of NF-κB (cat #8242), c-Jun (cat #9165), H3K9 (cat #9649), H3K18 (cat #13998), Histone H3 (Positive IP control, cat #: 4620 and Normal Rabbit IgG (Negative IP control, cat #2729), for overnight at 4 °C and incubated with Protein G magnetic beads for 2 h at 4 °C. Chromatin was eluted from Antibody/Protein G magnetic beads complex by incubation at 65 °C for 30 min using magnetic separation. Chromatin was reverse crosslinked by treating with proteinase K for 2 h at 65 °C and the DNA was purified from the ChIP fraction using spin column method. To be considered a true association, each ChIP sample was examined for the enrichment of a chromatin locus immunoprecipitated with a specific antibody and compared with the same chromatin locus immunoprecipitated with a non-specific IgG (ANOVA with *p* < 0.05). Data represent the mean ± SD from three independent biological experiments. The enrichment of DNA sequence was then detected by RT-qPCR using Syber green mix and Epitect qPCR primers. The primers flanking specific the transcription factor binding sites at both the proximal and distal Il6 gene promotor regions and were designed using https://www.ncbi.nlm.nih.gov/tools/primer-blast/ (accessed on 7 May 2024). Primers are listed in Table 1.

### 4.5. Western Blotting

3T3-L1 preadipocytes were incubated for 30 min with lysis buffer containing Tris (62.5 mM; pH 7.5), 1% Triton X-100, and 10% glycerol. Cell lysates were centrifuged at 14,000 rpm for 10 min, supernatants were collected, and protein was measured using Quick Start Bradford 1× Dye Reagent and a protein assay kit (Bio-Rad, Hercules, CA, USA). Samples (20 μg) were mixed with loading buffer, heated for 5 min at 95 °C, and resolved by 12% SDS-PAGE. Resolved proteins were transferred to an Immun-Blot PVDF Membrane (Bio-Rad) by electroblotting, blocked with 5% nonfat milk in PBS for 1h, and incubated overnight at 4 °C with primary Abs (1:1000 dilution; Cell Signaling Technology Inc., Danvers, MA, USA) against H3K9ac (cat #9649), H3K18ac (cat #13998). Blots were washed three times with TBST and incubated for 1h with HRP-conjugated secondary Ab (Promega, Madison, WI, USA). Immunoreactive bands were developed using an Amersham ECL Plus Western Blotting Detection System (GE Healthcare, Buckinghamshire, UK) and visualized using a Molecular Imager VersaDoc MP imaging system (Bio-Rad, Hercules, CA, USA).

### 4.6. Statistical Analysis

Data analysis was performed using GraphPad Prism software (La Jolla, CA, USA). Data are shown as mean ± SE of the mean. Unpaired Student t-tests and one-way ANOVA followed by Tukey’s test were used to compare means between groups. For statistical analysis, data from a minimum of three sample sets were used for statistical calculation, and a *p*-value < 0.05 was considered significant. NS: non-significant, * *p* < 0.05, ** *p* < 0.01, *** *p* < 0.001 and **** *p* < 0.0001).

## 5. Conclusions

Taken together, these data support a cooperativity model wherein TNF-α/stearate interaction upregulates IL-6 expression in 3T3-L1 adipocytes through the epigenetic remodeling hinged on H3K9/18 acetylation, and these findings may have implications for adipose inflammation in obesity.

## Figures and Tables

**Table 1:** List of primers for qPCR.

No	Primer	Sequence
1	IL6-distal-F	AACTGATAAAAAGGAAGGGAGGT
2	IL6-distal-R	CCTCTCTCTGTGGGGTTGAT
3	IL6-proximal-F	TAGGGCTAGCCTCAAGGATG
4	IL6-proximal-R	AGGAAGGGGAAAGTGTGCTT

## Data Availability

All data generated or analyzed during this study are included in this published article.

## Supplementary Materials

The following supporting information can be downloaded at: https://www.mdpi.com/article/10.3390/ijms25126776/s1.
