# Supplementary material for: TNF-α/Stearate Induced H3K9/18 Histone Acetylation Amplifies IL-6 Expression in 3T3-L1 Mouse Adipocytes
Source: Int J Mol Sci. 2024 Jun 20;25(12):6776. doi: 10.3390/ijms25126776 (PMC11203872; doi:10.3390/ijms25126776)
Supplement: Supplementary file 1 [file ijms-25-06776-s001.zip › ijms-3026151-supplementary.pdf]

Supplementary File

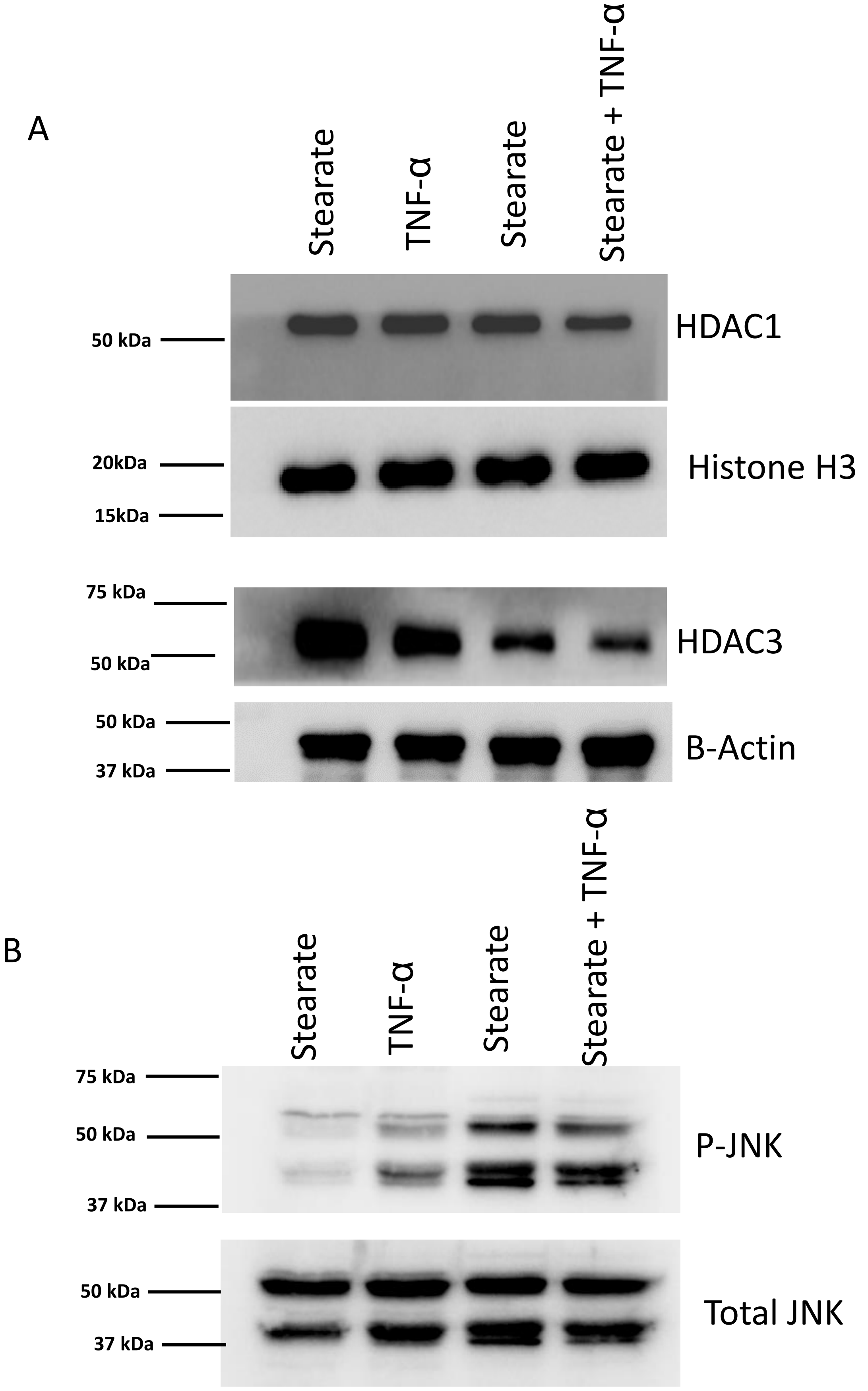

Figure S1: Impact of TNF-α, Stearate or Stearate/TNF-α on P-JNK , HDAC1 and HDAC3.

(A-B) 3T3-L1 preadipocytes were incubated for 30 min with lysis buffer containing Tris (62.5 mM; pH 7.5), 1% Triton X-100, and 10% glycerol. Cell lysates were centrifuged at 14,000 rpm for 10 min, supernatants were collected, and protein was measured using Quick Start Bradford 1× Dye Reagent and a protein assay kit (Bio-Rad, Hercules, CA). Samples (20 μg) were mixed with loading buffer, heated for 5 min at 95°C, and resolved by 12% SDS-PAGE. Resolved proteins were transferred to an Immun-Blot PVDF Membrane (Bio-Rad) by electroblotting, blocked with 5% nonfat milk in PBS for 1h, and incubated overnight at 4°C with primary Abs (1:1000 dilution; Cell Signaling Technology Inc., Danvers, MA, USA) against HDAC1 (Cat#2062), HDAC3 (Cat #85057), Histone H3 (Cat #4499), β-Actin (Cat #4967), PSAPK/JNK (cat #4668). Blots were washed three times with TBST and incubated for 1h with HRP-conjugated secondary Ab (Promega, Madison, WI). Immunoreactive bands were developed using an Amersham ECL Plus Western Blotting Detection System (GE Healthcare, Buckinghamshire, U.K.) and visualized using a Molecular Imager VersaDoc MP imaging system (Bio-Rad).
